# The Effect of Raw Sugar Addition on Flavor and Retronasal Olfaction Profiles of Processed Brown Sugar

**DOI:** 10.3390/foods14091480

**Published:** 2025-04-24

**Authors:** Yonathan Asikin, Yuki Nakaza, Moena Oe, Eriko Arakaki, Goki Maeda, Hirotaka Kaneda, Kensaku Takara, Koji Wada

**Affiliations:** 1 Department of Bioscience and Biotechnology, Faculty of Agriculture, University of the Ryukyus, Nishihara 903-0213, Japan; 2 United Graduate School of Agricultural Sciences, Kagoshima University, Kagoshima 890-0065, Japan; 3 Regional Agricultural System Section, Okinawa Prefectural Agricultural Research Center, Itoman 901-0336, Japan; 4 Okinawa Prefectural Agricultural Research Center Nago Branch, Nago 905-0012, Japan; 5 Department of Life Science, Faculty of Life Science, Kyushu Sangyo University, Fukuoka 813-8503, Japan

**Keywords:** processed brown sugar, raw sugar, flavor components, retronasal olfaction, Maillard reaction products, in-mouth aroma release

## Abstract

Processed brown sugar is produced by combining non-centrifugal cane sugar (NCS), raw sugar, and molasses. The present study aimed to examine the effects of NCS and raw sugar blending (10%:90%, 50%:50%, 75%:25%, and 90%:10%) on color traits, non-volatile and volatile compounds, retronasal aroma release, and sensory profiles of processed brown sugar, and hence, its flavor quality. The International Commission for Uniform Methods of Sugar Analysis (ICUMSA) color index and the +L* (brightness) and +b* (yellowness) color spaces were gradually altered upon the addition of raw sugar, with strong Pearson’s negative correlations between the ICUMSA value and both color space indices (r = −0.9554 and r = −0.9739, respectively), causing a lighter color of the final product. Raw sugar addition also significantly reduced the concentration of non-volatile compounds, such as glucose and organic acids (*p* < 0.05). As the raw sugar proportion increased from 10 to 90%, the concentrations of total volatile compounds and Maillard reaction products (MRPs), such as pyrazines, furans, and furanones, also decreased significantly from 62.58 to 22.73 µg/100 g and 34.75 to 6.80 µg/100 g, respectively. Reduced intensities of ion masses of in-mouth and in-nose retronasal odors from volatile MRPs, as well as roasted aroma and richness properties, were observed in processed brown sugars with greater raw sugar content. Taken together, a higher proportion of raw sugar in processed brown sugar manufacturing enhances brightness while reducing acidity and aftertaste; however, increased NCS content results in darker products with greater roasted aroma and richness, affecting flavor quality.

## 1. Introduction

Processed brown sugar is typically prepared by blending raw or refined sugar with molasses and other substances, allowing manufacturers to maintain product quality and economical production costs [1]. Conventional non-centrifugal cane sugar (NCS) is an unrefined, solidified form of sugarcane syrup without molasses removal, which makes it rich in minerals, amino acids, phenolics, and other bioactive compounds, as well as various aroma compounds [1,2,3]. Some of the important aroma-contributing compounds in NCS are volatile Maillard reaction products (MRPs), which are generated from non-enzymatic reactions between reducing sugars and amino acids in the condensed syrup during heating and evaporation processes [4,5]. NCS (*kokuto*) is widely used as an essential component in the production of processed brown sugar (*kako-kokuto*) in Okinawa, Japan, resulting in the retention of some characteristics of traditional NCS in the finished product [1,6].

Flavor, an indispensable quality of food, is highly influenced by its composition, which in turn affects its physical, chemical, and sensory properties [7,8]. Color traits, which are important determinants of the sensory perception of food products, are imparted by natural pigments or colorant compounds generated during processing [9]. For the NCS color, the International Commission for Uniform Methods of Sugar Analysis (ICUMSA) value is a reliable color index, using the reference method initially used for evaluating sugar impurities and refinement degrees in the sugar industry [5,10]. On the other hand, non-volatile and volatile substances play essential roles in determining the taste and aroma characteristics of foods, respectively, and to some extent, both components may be intertwined to influence sensory perception and acceptability [11,12]. The interactions between these compounds, along with their stability, release mechanisms, and matrix effects in food systems, considerably affect taste attributes such as sweetness, umami, bitterness, and overall palatability [2,12,13]. A thorough understanding of the chemical and physiological mechanisms that determine flavor quality is essential to optimize food formulations, enhance consumer acceptance, and develop innovative products with greater sensory appeal [14].

Retronasal olfaction, a dynamic sensory process involving volatile compounds released during mastication and swallowing, has a major impact on flavor perception, and its profile may be modified by several factors, such as food composition and texture, as well as oral processing behavior [13,15,16]. Real-time analytical techniques for assessing the retronasal olfaction profile of food products, particularly their aroma release, are crucial for determining which volatile substances have the greatest influence on perceived sensory preferences [15,16,17]. One of the most widely applied instruments for this purpose is the proton transfer reaction (PTR) system coupled with time-of-flight mass spectrometry (TOF-MS) or other types of MS detection systems [16,17,18]. The chemical reaction principle involves colliding the soft ionization of volatile compounds from ingested foods using proton transfer reactions with hydronium ions (H_3_O^+^) to generate protonated selective ion masses for monitoring released volatile compounds from panelists’ noses or mouths [17,19]. Volatile MRPs, such as pyrazines, furans, furanones, and pyrroles, have been identified as aroma-active compounds with desired distinct aromas that contribute to the retronasal olfaction profile of Okinawan conventional NCS [5,17]. The released aromas from the retronasal cavity during NCS consumption are positively associated with its sensory attributes and thus flavor characteristics [17]. Therefore, retronasal olfaction is a key consideration in determining the flavor quality of NCS products, including processed brown sugar.

Various studies have been undertaken to assess the physicochemical properties, nutrients, and volatile components of conventional NCS [2,3,5,17]. However, there is currently little information available on the flavor quality of processed brown sugar or the effect of combining NCS and raw sugar syrups on the physicochemical characteristics, flavor components, and retronasal aroma release profiles of the finished products. Therefore, the current study primarily aimed to evaluate the impact of raw sugar addition on the color traits and flavor quality of processed brown sugar. The study investigated the ICUMSA values, L*a*b* color spaces, non-volatile components, volatile aroma compounds, retronasal aroma release, and sensory profiles of processed brown sugar with different NCS-raw sugar blending ratios. To the best of our knowledge, this is the first report on the flavor and retronasal olfaction profiles of processed brown sugar supplemented with raw sugars. This study revealed the influence of blending raw sugar and NCS to manufacture new types of brown sugar products with adjustable quality traits while retaining some of the characteristics of traditional NCS.

## 2. Materials and Methods

### 2.1. Standards and Reagents

2,2-Dimethyl-2-silapentane-5-sulfonate (DSS) sodium salt, 1,2-dichlorobenzene-D4, and an alkane mixture (C_7_–C_30_) were obtained from Sigma Aldrich (St. Louis, MO, USA). Deuterium oxide (D_2_O) was purchased from Cambridge Isotope Laboratories (Andover, MA, USA). Methanol (LC-MS grade) was purchased from Fujifilm Wako Pure Chemical Industries (Osaka, Japan). All the other reagents were of analytical grade.

### 2.2. Processed Brown Sugar Production Using Tabletop Manufacturing Equipment

The processed brown sugar model with added raw sugar was produced using tabletop equipment with continuous heating and agitation devices (Nishikawa Keisoku, Tokyo, Japan) [6]. Sugar syrup was made with NCS powder of Hateruma Island origin (Lot No. 21117) provided by the Okinawa Prefecture Brown Sugar Cooperative Association, and raw sugar was purchased from a local market (Aoiumi Co., Ltd., Okinawa, Japan). The syrup mix was prepared by combining NCS and raw sugar syrups at 50% Brix in the following ratios: 10%:90%, 50%:50%, 75%:25%, and 90%:10% (*w*/*w*). The syrup (800 g) was fed into the tabletop manufacturing equipment and evaporated from 25 °C to a final heating temperature of 130 °C with constant agitation at 100 rpm. Subsequently, the condensed syrup was solidified without heat in an aluminum tray to yield approximately 300 g of brown sugar. The solidified sugar was ground into a powder. All experiments were performed in four replicates. The brown sugar was kept at −30 °C before analysis.

### 2.3. Color Traits Analysis Using L*a*b* Color Spaces and ICUMSA Value Determination

Briefly, 10 g of processed brown sugar was dissolved thoroughly in ultrapure water and then made up to a 20% (*w*/*v*) solution in a 50 mL volumetric flask. The mixture was then centrifuged at 3000 rpm for 10 min. The L*a*b* color spaces of the supernatants were measured using a CM-2600d spectrophotometer (Konica Minolta, Tokyo, Japan). The concentration of the aqueous brown sugar solution was adjusted to 4%, the soluble solids were measured using a portable refractometer (RePo; Atago, Tokyo, Japan), and the absorbance at 420 nm was measured using an SH-9000La spectrophotometer (Corona Electric, Ibaraki, Japan) [5]. The ICUMSA value (IU) was determined using the equation IU = 1000 × (100 × *E*)/(*d* × *Bx* × *D*), where *E* is the absorbance at 420 nm, *d* is the width of the cell (cm), *Bx* is %Brix, and *D* is the solution density. All assays were performed in triplicate.

### 2.4. Non-Volatile Components Analysis Using ^1^H Nuclear Magnetic Resonance

The non-volatile components of the processed brown sugars, such as sugars, organic acids, and amino acids, were examined using non-targeted ^1^H nuclear magnetic resonance (NMR) [2]. Briefly, 140 µL of 20% (*w*/*v*) processed brown sugar solution and 560 µL of 1 mM DSS as the internal standard (prepared in 500 mM potassium phosphate buffer pH 7.0 in D_2_O) were mixed and placed into a 5 mm-diameter NMR tube. ^1^H-NMR spectra were acquired using a Bruker Avance 500 MHz NMR spectrometer (Bruker BioSpin, Karlsruhe, Germany) with the zgpr pulse program from the Bruker pulse library in digital quadrature detection mode. The spectra were collected using 64 scans and a 4 s relaxation delay. The offset frequency of the protons was at 4.7 ppm and the 90° pulse width used was 12 μs. The compound signals were annotated, and the relative concentrations of the compounds were normalized to the internal standard using the Chenomx NMR Suite Version 8.6 (Chenomx Inc., Edmonton, AB, Canada). All assays were performed in triplicate.

### 2.5. Volatile Components Analysis Using Solid-Phase Microextraction Gas Chromatography MS

The volatile components of the processed brown sugar were analyzed using solid-phase microextraction (SPME) gas chromatography (GC) MS [17]. Briefly, 3 g of processed brown sugar and 20 µL of 2.5 µg/mL 1,2-dichlorobenzene-D4 (prepared in methanol) as the internal standard were placed into a 20 mL vial and heated at 60 °C for 5 min in a CombiPAL autosampler (CTC Analytics, Zwingen, Switzerland). Volatiles in the headspace were extracted using a divinylbenzene/carboxen/polydimethylsiloxane 50/30 µm SPME fiber (Supelco, Bellefonte, PA, USA) at 60 °C for 20 min under continuous agitation. The SPME fibers were then desorbed using an Agilent 7890 B GC (Agilent Technologies, Santa Clara, CA, USA) at a split ratio of 10:1. Helium was used as the carrier gas at a linear velocity of 21 cm/s on a DB-Wax column (30 m × 0.25 mm, 0.25 µm; Agilent Technologies). The oven temperature was initially set at 40 °C for 1 min, increased to 200 °C at 3 °C/min, and finally held at 200 °C for 17 min. MS spectra were acquired using an Agilent 5977A MSD at *m*/*z* 33–450 in electron ionization mode at 70 eV. The temperature of the ion source and transfer line were kept at 230 °C. Volatile compounds were identified based on linear retention index (RI) comparisons (<|20|) and similarities with MS data obtained from the NIST Library Version 17 (>80%). The weight intensity of the peak areas was calibrated to the internal standard response, and compound concentrations were expressed in µg/100 g. All assays were performed in triplicate.

### 2.6. Retronasal Olfaction Profile Analysis Using PTR-TOF-MS

The in-nose and in-mouth aroma released upon intake of the processed brown sugar solution was analyzed using PTR-TOF-MS [17]. Ten panelists (three males and seven females, aged 22–61 years) were asked to drink 20 mL of processed brown sugar solution (15% *w*/*v*), and the volatile compounds in their breath were recorded on a PTR-TOF 1000 ULTRA instrument (Ionicon Analytik, Innsbruck, Austria) through a capillary inlet hose and nose- and mouth-piece adapters. The intensity of representative MS ions of the volatile compounds was detected per second at a detection limit of 5 ppt⋅V. Ionization was carried out at an E/N ratio of 122 Td, and the drift temperature, voltage, and pressure were set to 120 °C, 479.9 V, and 2.30 mbar, respectively. The panelists were asked to collect 20 mL of water from a disposable paper cup, hold it in their mouth for 5 s, and swallow it. They were then required to put on a nosepiece adapter and exhale for 2 s from the nose, and then inhale through the nose for 2 s. The breathing cycle was repeated seven times. Following a 10 s interval, the panelists were instructed to take 20 mL of brown sugar solution and breathe out through their noses seven times. They were then asked to rest for 5 min and rinse their palates with water by gargling five times to remove any residual brown sugar solution from their mouths. The same procedure was repeated to measure in-mouth aroma release, with panelists taking water or the brown sugar solution and finally breathing out through their mouths into a mouthpiece adapter. The room temperature was maintained at 25 °C throughout the experiments. This study was approved by the Research Ethics Committee of Kyushu Sangyo University (No. 2022-0003) and verbal consent was obtained from all participants prior to the experiment.

### 2.7. Evaluation of Sensory Properties

The sensory properties of the processed brown sugar were analyzed using an unstructured line-scale method. Ten panelists (the same panelists participated in the PTR-TOF-MS analysis) were asked to evaluate 20 mL of processed brown sugar solution (15% *w*/*v*) supplied in disposable paper cups labeled with three-digit random numbers on an unstructured 10 cm scale anchored with the highest or lowest intensity of the following sensory evaluation criteria: sweet aroma, roasted aroma, sweetness, bitterness, richness, and aftertaste. Water was provided to the panelists to rinse their palates before each sample was evaluated.

### 2.8. Statistical Analysis

Statistical differences among mean values were analyzed using Tukey’s multiple comparisons test. Correlations between two parameters were examined using Pearson’s correlation analysis (GraphPad Prism Version 9; GraphPad Software, San Diego, CA, USA).

## 3. Results

### 3.1. Color Properties of Processed Brown Sugar

**Figure 1:**
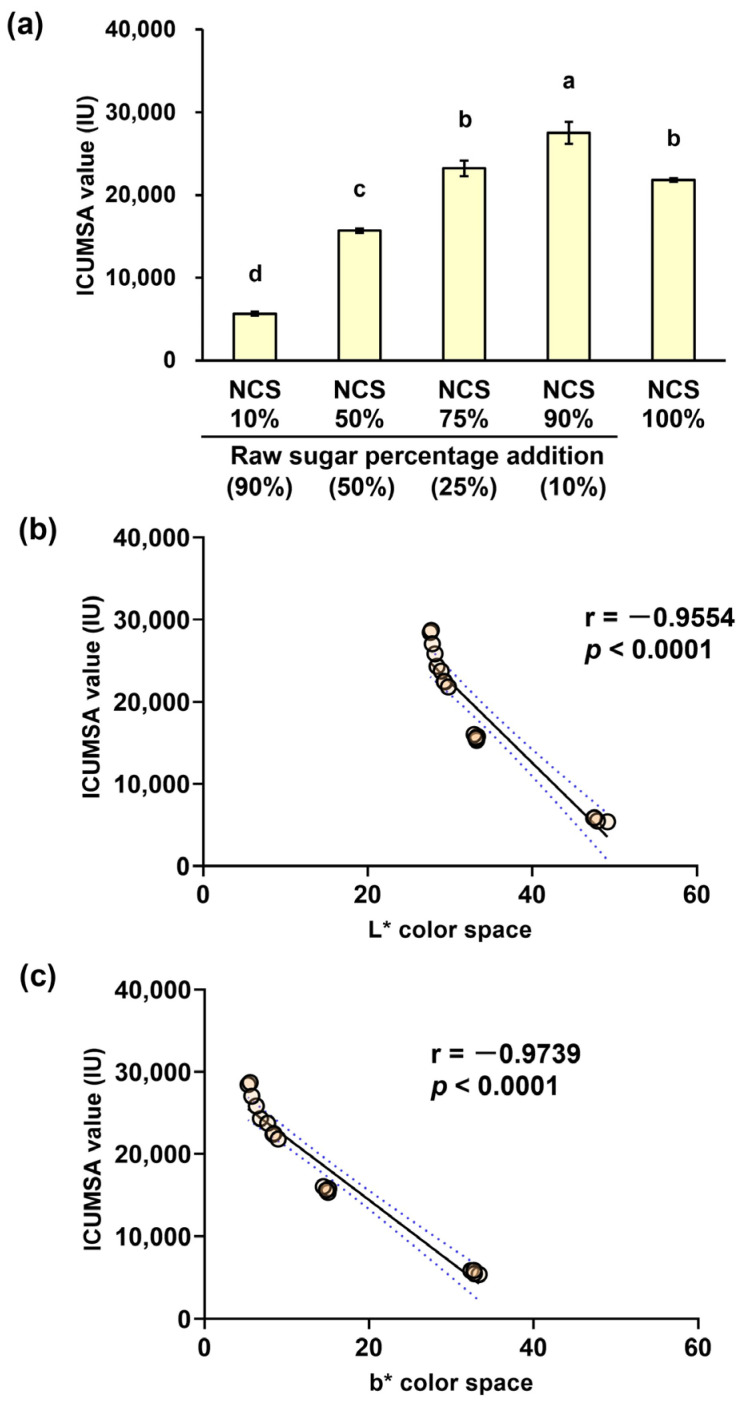
(**a**) International Commission for Uniform Methods of Sugar Analysis (ICUMSA) value of processed brown sugars with different percentages of raw sugar addition (each value is expressed as the mean ± standard deviation of four replicates; means followed by the different letters are significantly different at *p* < 0.05; NCS, non-centrifugal cane sugar); (**b**) Pearson’s correlation between the ICUMSA value and the +L* color space; (**c**) Pearson’s correlation between the ICUMSA value and the +b* color space.

The ICUMSA value of processed brown sugar with 90% NCS content (or 10% raw sugar addition) was significantly greater than that of conventional NCS (NCS 100%) and other processed brown sugars with lower NCS concentrations, that is, 10, 50, and 75% NCS content (*p* < 0.05) (Figure 1a). This value gradually decreased as the proportion of raw sugar increased, with the value of the product with 90% NCS content being 4.86-fold higher than that of the product with 10% NCS content (27,500 vs. 5658 IU). Likewise, there were gradual changes in color attributes, such as +L* (brightness) and +b* (yellow) color spaces, as the percentage of added raw sugar increased (Supplementary Table S1). Thoroughly, the +L* color space value of processed browns sugar product significantly increased from 27.84 to 48.02 as raw sugar percentage addition increased from 10 to 90%, whereas processed brown sugar with 90% NCS content (10% raw sugar addition) had a significantly lower +b* value than those with higher raw sugar percentages (5.73 vs. 7.81–32.83) (*p* < 0.05). Moreover, these color attributes were significantly negatively correlated with the ICUMSA values (Pearson’s correlation coefficients r = −0.9554 and r = −0.9739, respectively; *p* < 0.0001), indicating that the addition of raw sugar in the processed brown sugar could reduce the ICUMSA color intensity while increasing the brightness and yellowness of the final products (Figure 1b,c).

### 3.2. Non-Volatile Components of Processed Brown Sugar

Seven non-volatile substances were detected in conventional NCS (NCS 100%) and processed brown sugars using ^1^H-NMR analysis, including two sugars (sucrose and glucose), four organic acids (trans-aconitate, formate, acetate, and succinate), and an amino acid (threonine) (Table 1). The addition of raw sugar to processed brown sugar altered the concentration of its non-volatile components. There was no significant difference in sucrose concentration between conventional NCS and any of the processed brown sugars (982.35–1099.27 mM). There was no significant difference in glucose concentration between processed brown sugar products with up to 50% raw sugar addition (*p* < 0.05). However, the concentration of glucose in processed brown sugar with 10% NCS was significantly lower than that in brown sugar with 75% NCS content (36.40 vs. 60.23 mM, respectively). Moreover, the concentrations of threonine and organic acids gradually decreased as the percentage of raw sugar increased, with significantly lower concentrations observed in the processed brown sugar with lower NCS contents, that is, 10 and 50% NCS.

### 3.3. Volatile Components of Processed Brown Sugar

**Figure 2:**
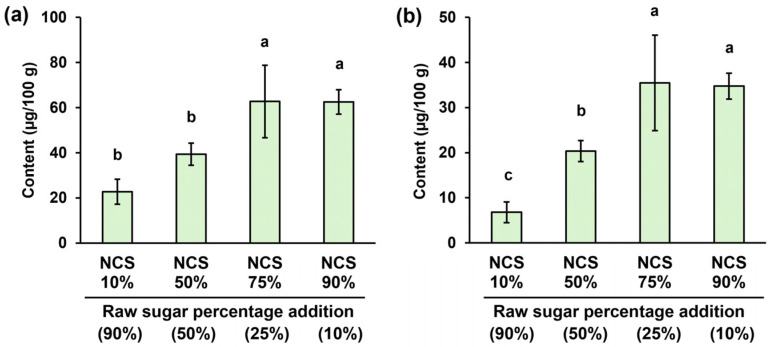
Total concentrations of (**a**) volatile organic components and (**b**) Maillard reaction products of processed brown sugars with different percentages of raw sugar addition. Each value is expressed as the mean ± standard deviation of four replicates. Means in the same group followed by the different letters are significantly different at *p* < 0.05. NCS, non-centrifugal cane sugar.

The total content of volatile components in the processed brown sugar gradually decreased as the proportion of raw sugar increased (Figure 2a). There were fewer total volatile compounds in the conventional NCS than in the processed brown sugar with 90% NCS content. This phenomenon occurred because of the accumulation of volatile compounds from the ingredients, combined with the generation of additional volatile compounds in the processed brown sugar as a result of the reheating procedure used in its manufacturing. The total volatile compound concentrations in processed brown sugars with 75 and 90% NCS content were 62.73 and 62.58 µg/100 g, respectively, while products with 10 and 50% NCS content had significantly lower levels (*p* < 0.05). A similar gradual decline trend was observed for the total volatile MRP concentrations as the proportion of raw sugar increased (Figure 2b). The highest total MRP concentration was found in processed brown sugars with 75 and 90% NCS content (35.47 and 34.75 µg/100 g, respectively), followed by products with 50% NCS content (20.33 µg/100 g) and products with 10% NCS content (6.80 µg/100 g). The volatile MRPs in the processed brown sugars included pyrazines (pyrazine, 2-methylpyrazine, 2,5-dimethylpyrazine, 2,6-dimethylpyrazine, 2-ethylpyrazine), furans (2-pentylfuran, 3-formylfuran, furfural, 3-formylfuran, 5-methyl-2-furanmethanol), and furanones (2-methyldihydro-3(2H)-furanone, dihydro-2(3H)-furanone, 2(5H)-furanone, 3-hydroxy-4,4-dimethyldihydro-2(3H)-furanone) (Supplementary Table S2). Most of these aroma compounds decreased in concentration as the proportion of NCS decreased or raw sugar increased. However, the concentrations of other volatile compounds in the processed brown sugars varied arbitrarily.

### 3.4. Retronasal Olfaction and Sensory Profiles of Processed Brown Sugar

**Figure 3:**
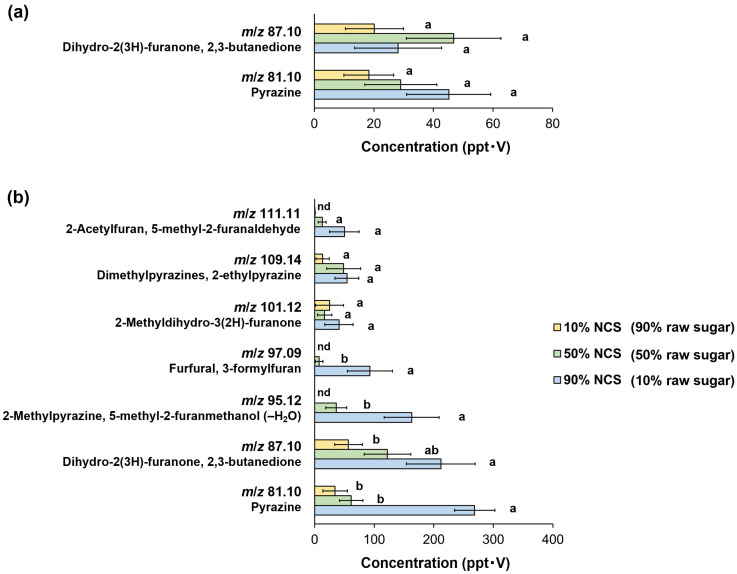
Retronasal olfaction profiles of volatile Maillard reaction products of processed brown sugars with different percentages of raw sugar addition. (**a**) In-nose aroma release. (**b**) In-mouth aroma release. Each value is expressed as the mean ± standard error of 10 panelists. Means in the same group followed by the different letters are significantly different at *p* < 0.05. NCS, non-centrifugal cane sugar.

The intensity of in-nose and in-mouth retronasal odors induced by consuming processed brown sugar gradually decreased as the proportion of raw sugar increased (Figure 3). Two ion masses, *m*/*z* 81.10 and 87.10, were detected from the in-nose aroma release following processed brown sugar consumption (Figure 3a). Although the intensity of the ion mass *m*/*z* 81.10 (protonated pyrazine) increased from 18.31 to 45.15 ppt⋅V in processed brown sugar with 10% and 90% NCS contents, respectively, there was no significant difference when they were consumed. In contrast, no trend was observed in the intensity of the ion mass *m*/*z* 87.10 (protonated dihydro-2(3H)-furanone or 2,3-butanedione). Furthermore, seven ion masses were discovered in in-mouth retronasal aroma, with the ion mass *m*/*z* 81.10 being the strongest detectable signal in processed brown sugar with 90% NCS content, at 268.65 ppt⋅V, followed by ion masses *m*/*z* 87.10, 95.12, and 97.09 (211.82, 162.95, and 92.94 ppt⋅V, respectively) (Figure 3b). The intensities of these four ion masses from the in-mouth aroma release were significantly higher for processed brown sugar (90% NCS content) than products with a lower NCS content or higher raw sugar proportion (*p* < 0.05). However, there was no significant change in the detection of the remaining three ion masses (*m*/*z* 101.12, 109.14, and 111.11) across all processed brown sugars, even though their intensities gradually rose as the NCS proportion increased. Additionally, no signals were found for ion masses *m*/*z* 95.12, 97.09, or 111.11, after ingesting the product with 10% NCS content.

**Figure 4:**
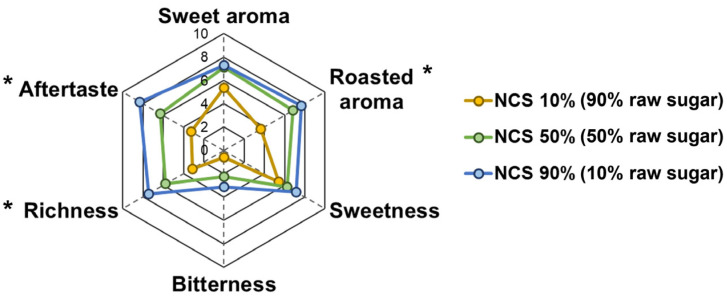
Sensory properties of processed brown sugars with different percentages of raw sugar addition. The notations of significant differences between samples are as follows: * non-centrifugal cane sugar (NCS) 10%, b; NCS 50%, a; NCS 90%, a (different letters indicate significant differences at *p* < 0.05).

There were gradual changes in the perceived sensory characteristics of processed brown sugar with different percentages of raw sugar, with products with a higher NCS content (or lower raw sugar proportion) being richer in all the evaluated taste and aroma traits (Figure 4). The roasted aroma was assessed at significantly higher rates in processed brown sugar with 50 and 90% NCS content (average values: 6.83 and 7.68 out of 10.00, respectively) than in the product with 10% NCS content (3.66) (*p* < 0.05). Significant differences were also observed in the richness and aftertaste attributes (5.76–7.43 vs. 3.14 and 6.29–8.31 vs. 3.25, respectively). The addition of raw sugar at a concentration of 90% to the processed brown sugar significantly diminished its roasted aroma, richness, and aftertaste. However, no significant changes were found for sweet aroma, sweetness, or bitterness characteristics. Therefore, a high proportion of raw sugar in processed brown sugar reduced its detectable aftertastes, while a higher NCS content enhanced roasted aroma and richness.

## 4. Discussion

Traditional NCS contains unseparated molasses that has been concentrated to form a brown color on the solidified sugar, followed by Maillard reactions and caramelization, which can intensify the dark brown color during thermal processes, such as heating and evaporation [1,3,4,20]. The inclusion of raw sugar in the manufacture of processed brown sugar considerably affects the color properties of the finished product. Raw sugar addition reduces the browning appearance of the processed brown sugar product because it contains fewer concentrated molasses and colorant compounds such as melanoidins than traditional NCS [1,4]. Alterations in the ICUMSA value, a key indicator of sugar color intensity, as well as the brightness (+L) and yellowness (+b) indices, thus emphasize the importance of conventional NCS and raw sugar proportion balances in the inverse relationship between product brightness and color intensity. The impact of these color differences, combined with the negative correlations between the ICUMSA value and yellowness index in the processed brown sugar products, extends beyond its simple physical trait, as color is a critical sensory indicator that influences sensory experiences and perceptions, with darker brown sugar perceived to have a richer flavor quality, which can increase consumer appeal [21,22].

The inclusion of raw sugar in the manufacturing of processed brown sugars altered the composition of non-volatile components, particularly the amounts of glucose, threonine, and organic acids, as expected, given the reduced percentage of conventional NCS in their constituents. The relatively stable sucrose levels across all processed brown sugar products suggested that the addition of raw sugar had little influence on this predominantly non-volatile component. A notable reduction in the concentration of glucose and amino acids, such as threonine, may be attributed to the occurrence of non-enzymatic Maillard reactions that generate volatile MRPs with pleasant aromas [2,23]. However, in the present study, the lack of conventional NCS as the primary source of these substances was the main cause of deficits in processed brown sugar with higher raw sugar content. Additionally, a reduction in the amount of organic acids due to a higher raw sugar content might affect the taste characteristics of the final products, as organic acids such as acetic and succinic acids may provide unique acidic and tangy notes that balance sweetness and enhance the overall flavor quality of brown sugar products [2,24].

The proportion of raw sugar in processed brown sugar also greatly affects its volatile compound composition, as evidenced by a decrease in the total concentration of volatile compounds with increasing raw sugar content, potentially diminishing its odor strength. Similarly, the concentration of volatile MRPs decreased in processed brown sugars with higher raw sugar levels (or lower conventional NCS proportions). The lack of concentration of reducing sugar substances in raw sugar, which are precursors to Maillard reactions, could be the primary cause of this occurrence [2,3]. Previous studies have reported that some of these MRPs are recognized as aroma-active compounds in NCS products, making them important for sensory complexity and quality because each compound has desirable pleasant aroma characteristics [5,25]. For example, pyrazines provide roasted and nutty aromas, whereas furans and furanones emit sweet, maple, and caramel-like odors [5,25,26]. Therefore, the addition of raw sugar could compromise the formation of key aromatic compounds that downgrade the sensory quality of the processed brown sugar.

The retronasal olfaction profiles of processed brown sugar have revealed that the ingredients modulate aroma release during consumption, affecting both in-nose and in-mouth odor sensations. The enhanced in-mouth aroma release showed that a higher NCS proportion (or lower raw sugar addition) in the product promoted the release of volatile MRPs, such as pyrazines and furanones, during mastication. As expected, only a few volatile compounds (pyrazine [*m*/*z* 81.10] and dihydro-2(3H)-furanone and 2,3-butanedione [*m*/*z* 87.10]) were able to reach the panelists’ nose cavities as an in-nose aroma release. These molecules could deliver nutty, sweet, and buttery scent signals to the olfactory receptors, respectively [5,26], and they were perceived. Furthermore, variations in in-mouth retronasal intensities were typically correlated with the concentration of corresponding volatiles in processed brown sugars, which increased as NCS content increased (Supplementary Table S2). However, the trend was not discernible in a number of odor signals, i.e., *m*/*z* 101.12, 109.14, and 111.11, where the concentrations of the compounds related to the ion masses, including 2-methyldihydro-3(2H)-furanone, 2,5-dimethylpyrazine, 2,6-dimethylpyrazine, 2-ethylpyrazine, 2-acetylfuran, and 5-methyl-2-furanaldehyde, were found to be relatively low. This suggests that their contributions to the released aromas might be considered background odor notes rather than being particularly impactful. Therefore, the in-nose release of volatile compounds serves as an initial primer for sensory expectation, whereas in-mouth release is critical for sustaining flavor complexity and intensifying the sensory experience of consumed products [15,16,17].

Processed brown sugar with a higher conventional NCS content exhibited an improved roasted aroma and richness, which may promote its overall flavor quality. These sensory traits might be influenced by volatile MRPs and taste-contributing substances, respectively [17,25,27]. Conversely, the products have a stronger aftertaste than processed brown sugar with the lowest NCS content (90% raw sugar proportion), which may reduce taste acceptance. However, aftertaste, or the opposing trait, a “clean” finish taste, maybe a necessary sensory quality for brown sugar, and it should be further explored in future hedonic studies. Future investigations should also explore the interaction between non-volatile and volatile components on sensory acceptability for a better understanding of flavor dynamics in brown sugar. Nevertheless, the present study established a solid foundation for the variable sensory qualities of processed brown sugars. Overall, these findings underscore the potential of optimizing raw sugar addition in processed brown sugar manufacturing to adjust the flavor quality. For example, a high proportion of raw sugar could be added to manufacture a processed brown sugar product with a lighter color, lower acidity, and less noticeable aftertaste, whereas a lower proportion of raw sugar could be supplemented to produce a final product with a darker color and stronger roasted aroma and richness, enabling effective product development strategies targeted at increasing consumer satisfaction and market competitiveness.

## 5. Conclusions

The addition of raw sugar to processed brown sugar caused a cascade of interconnected changes in physical, chemical, and sensory properties. A higher raw sugar proportion (lower conventional NCS content) resulted in a lighter and less intense brown color, indicating a lack of molasses in the product. This processed brown sugar also lacked total volatile compounds and key flavor precursors, such as glucose and amino acids, which promote the formation of volatile MRPs, such as pyrazines, furans, and furanones. The volatile compounds enhanced both in-nose and in-mouth retronasal aroma release and were strongly associated with enhanced sensory ratings of roasted aroma, richness, and sustained aftertaste. Sugar producers should pay attention to these intricate relationships that underline the critical balance between raw sugar and conventional NCS levels when developing processed brown sugars with optimal sensory appeal and consumer acceptance. This study also provides information to help industrial and individual buyers understand the expected quality when purchasing processed brown sugar products for use in food formulation and product development.

## Figures and Tables

**Table 1:** Concentrations of non-volatile components (mM) in processed brown sugars with different percentages of raw sugar addition.

Compound	NCS 10% (90% Raw Sugar)	NCS 50% (50% Raw Sugar)	NCS 75% (25% Raw Sugar)	NCS 90% (10% Raw Sugar)	NCS 100%
Sucrose	1002.38 ± 170.69 a	1099.27 ± 138.43 a	1073.97 ± 109.58 a	982.35 ± 51.61 a	992.41 ± 38.66 a
Glucose	36.40 ± 2.76 b	43.09 ± 11.57 ab	60.23 ± 11.98 a	57.28 ± 8.17 ab	37.23 ± 10.22 ab
Threonine	1.19 ± 0.15 c	3.89 ± 0.32 b	5.58 ± 1.03 a	6.46 ± 0.26 a	6.90 ± 0.46 a
trans-Aconitate	1.67 ± 0.23 d	2.95 ± 0.25 c	3.81 ± 0.67 bc	4.53 ± 0.74 b	6.55 ± 0.04 a
Formate	0.77 ± 0.07 c	1.45 ± 0.07 b	2.36 ± 0.21 a	2.72 ± 0.31 a	2.39 ± 0.25 a
Acetate	0.52 ± 0.05 d	1.75 ± 0.16 c	2.85 ± 0.41 b	3.38 ± 0.19 b	4.15 ± 0.29 a
Succinate	0.15 ± 0.02 d	0.44 ± 0.02 c	0.56 ± 0.04 b	0.67 ± 0.03 a	0.71 ± 0.05 a

Each value is expressed as the mean ± standard deviation of four replicates. Means in the same row followed by different letters are significantly different at *p* < 0.05. NCS, non-centrifugal cane sugar.

## Data Availability

The original contributions presented in the study are included in the article, further inquiries can be directed to the corresponding author.

## Supplementary Materials

The following supporting information can be downloaded at: https://www.mdpi.com/article/10.3390/foods14091480/s1, Table S1. L*a*b* color spaces of processed brown sugars with different percentages of raw sugar addition. Table S2. Volatile organic components of processed brown sugars with different percentages of raw sugar addition.

## References

[1] Ono H. (2020). Production and processing of brown sugar. J. Cook. Sci. Jpn..

[2] Liu J., Wan P., Zhao W., Xie C., Wang Q., Chen D.W. (2022). Investigation on taste-active compounds profile of brown sugar and changes during lime water and heating processing by NMR and e-tongue. LWT.

[3] Ge Y., Li K., Xie C., Xu Y., Shi C., Hang F., Doherty W.O.S. (2021). Formation of volatile and aroma compounds during the dehydration of membrane-clarified sugarcane juice to non-centrifugal sugar. Foods.

[4] Sung W.C., Chi M.H., Chiou T.Y., Lin S.H., Lee W.J. (2020). Influence of caramel and molasses addition on acrylamide and 5-hydroxylmethylfurfural formation and sensory characteristics of non-centrifugal cane sugar during manufacturing. J. Sci. Food Agric..

[5] Asikin Y., Hirose N., Tamaki H., Ito S., Oku H., Wada K. (2016). Effects of different drying–solidification processes on physical properties, volatile fraction, and antioxidant activity of non-centrifugal cane brown sugar. LWT Food Sci. Technol..

[6] Hirose N., Ono H., Maeda G., Wada K. (2019). Development of tabletop type manufacturing equipment for test production of non-centrifugal cane sugar “*Kokuto*” and the rise of syrup temperature during the cooling-agitation process of Kokuto production. Nippon Shokuhin Kagaku Kogaku Kaishi.

[7] Sung J., Frost S., Suh J.H. (2024). Progress in flavor research in food: Flavor chemistry in food quality, safety, and sensory properties. Food Chem. X.

[8] De Santis D. (2024). Food flavor chemistry and sensory evaluation. Foods.

[9] Singh T., Pandey V.K., Dash K.K., Zanwar S., Singh R. (2023). Natural bio-colorant and pigments: Sources and applications in food processing. J. Agric. Food Res..

[10] Puke H. (2017). VDZ and ICUMSA—125 years of scientific work for the sugar industry. Sugar Ind..

[11] Nothnagel T., Ulrich D., Dunemann F., Budahn H. (2023). Sensory perception and consumer acceptance of carrot cultivars Are influenced by their metabolic profiles for volatile and non-volatile organic compounds. Foods.

[12] Dubrow G.A., Tello E., Schwartz E., Forero D.P., Peterson D.G. (2022). Identification of non-volatile compounds that impact consumer liking of strawberry preserves: Untargeted LC-MS analysis. Food Chem..

[13] Lina G., Min Z. (2022). Formation and release of cooked rice aroma. J. Cereal Sci..

[14] Queiroz L.P., Nogueira I.B.R., Ribeiro A.M. (2024). Flavor Engineering: A comprehensive review of biological foundations, AI integration, industrial development, and socio-cultural dynamics. Food Res. Int..

[15] van Eck A., Pedrotti M., Brouwer R., Supapong A., Fogliano V., Scholten E., Biasioli F., Stieger M. (2021). In vivo aroma release and dynamic sensory perception of composite foods. J. Agric. Food Chem..

[16] Le Bechec M., Reyrolle M., Chesneau V., Breuil M., Desauziers V. (2024). Rapid analysis of VOCs with SIFT-MS as a decision-making support tool for caviar producers. Food Control.

[17] Asikin Y., Nakaza Y., Oe M., Kaneda H., Maeda G., Takara K., Wada K. (2024). Volatile component composition, retronasal aroma release profile, and sensory characteristics of non-centrifugal cane sugar obtained at different evaporation temperatures. Appl. Sci..

[18] Hayashi K., Nakada Y., Sémon E., Salles C. (2022). Retronasal aroma of beef pate analyzed by a chewing simulator. Molecules.

[19] Smith D., Španěl P., Demarais N., Langford V.S., McEwan M.J. (2025). Recent developments and applications of selected ion flow tube mass spectrometry (SIFT-MS). Mass Spectrom. Rev..

[20] Quintas M.A.C., Brandão T.R.S., Silva C.L.M. (2007). Modelling colour changes during the caramelisation reaction. J. Food Eng..

[21] Orlandi R.D.M., Verruma-Bernardi M.R., Sartorio S.D., Borges M.T.M.R. (2017). Physicochemical and sensory quality of brown sugar: Variables of processing study. J. Agric. Sci..

[22] Alves V., dos Santos J.M., Pinto E., Ferreira I.M.P.L.V.O., Lima V.A., Felsner M.L. (2024). Digital image processing combined with machine learning: A new strategy for brown sugar classification. Microchem. J..

[23] Wong K.H., Abdul Aziz S., Mohamed S. (2008). Sensory aroma from Maillard reaction of individual and combinations of amino acids with glucose in acidic conditions. Int. J. Food Sci. Tech..

[24] Shi Y., Pu D., Zhou X., Zhang Y. (2022). Recent progress in the study of taste characteristics and the nutrition and health properties of organic acids in foods. Foods.

[25] Liu J., Wan P., Xie C., Chen D.W. (2021). Key aroma-active compounds in brown sugar and their influence on sweetness. Food Chem..

[26] Chen E., Zhao S., Song H., Zhang Y., Lu W. (2022). Analysis and comparison of aroma compounds of brown sugar in Guangdong, Guangxi and Yunnan using GC-O-MS. Molecules.

[27] Gao Y., Cao Q.Q., Chen Y.H., Granato D., Wang J.Q., Yin J.F., Zhang X.B., Wang F., Chen J.X., Xu Y.Q. (2022). Effects of the baking process on the chemical composition, sensory quality, and bioactivity of Tieguanyin oolong tea. Front. Nutr..

